# Effects of short-form video app addiction on academic anxiety and academic engagement: The mediating role of mindfulness

**DOI:** 10.3389/fpsyg.2024.1428813

**Published:** 2024-10-09

**Authors:** Gongyu Li, Yaxuan Geng, Tingting Wu

**Affiliations:** ^1^School of Education, Minzu University of China, Beijing, China; ^2^School of Early Childhood Education, Hunan Vocational College for Nationalities, Yueyang, China; ^3^School of Early Childhood Education, Changsha Preschool Education College, Changsha, China

**Keywords:** short-form video app addiction, academic anxiety, academic engagement, mindfulness, academic performance

## Abstract

**Introduction:**

The impact of short-form video app addiction on academic performance, including academic anxiety and engagement, has not been adequately explored or addressed.

**Method:**

This study tested the effects of short-form video app addiction on academic anxiety and academic engagement and the mediating role of mindfulness in these relationships. The participants were 1,879 undergraduates in China who completed the Short-Form Video App Addiction Scale, Mindful Attention Awareness Scale, Academic Anxiety Sub-questionnaire of Academic Emotions Questionnaire, and Engagement Scales. Structural equation modelling (SEM) was used to analyse the data.

**Results:**

The results indicate that short-form video app addiction has significant positive effects on academic anxiety and negative effects on academic engagement. Furthermore, short-form video app addiction has significant indirect effects on academic anxiety and engagement via mindfulness. The proportions of the mediation effects are 19.23 and 51.85%, respectively.

**Discussion:**

The implications and limitations of this study are discussed.

## 1 Introduction

Short-form videos are a type of online video with a relatively short duration, typically between a few seconds and a few minutes (Dong et al., 2023). Recently, short-form video apps have increased in popularity (Qu et al., 2023), including but not limited to platforms such as TikTok, Kuaishou, and Huoshan. Taking TikTok as an example, as of December 2022, the number of users had exceeded 800 million, of which 700 million are daily active users, with an average daily use time per person of more than 2 h.^1^ Previous studies have found that short-form videos can have positive individual effects, including obtaining new knowledge and learning methods, possessing the ability to extract implicit knowledge that is difficult to obtain fully in real life, and bringing spiritual pleasure (Wang et al., 2023). As short-form video use continues to increase, short-form video addiction is gaining attention. Short-form video addiction is a subcategory of Internet addiction (Zhang et al., 2019) and mobile phone addiction and refers to an inability to control one’s short-form video app use, despite negative social and psychological consequences (Wang and Lei, 2022). People can become addicted to short-form video apps relatively easily (Zhang et al., 2019), which is due to several factors. First, personalized recommendation algorithms for short-form videos inundate users with information tailored to their individual preferences. Second, short-form videos provide users with stimulation and satisfaction during fragmented periods. Third, despite being rich in information, short-form videos are characterized by concise interactions and low cognitive engagement. Fourth, watching short-form videos allows users to escape stressful environments and alleviate negative emotions (Dong et al., 2023). Furthermore, some scholars have identified other factors that may contribute to short-form video addiction. For example, from sociotechnical and attachment perspectives, Zhang et al. (2019) noted that short-form video apps provide users with more opportunities to engage in interpersonal communication and make new friends, helping in the formation of interpersonal attachments through these apps. Unique technical features and functions can help users easily watch and upload various interesting short-form videos, which can also make them functionally dependent on these apps (Zhang et al., 2019).

Despite widespread recognition of the severely negative effects of short video addiction on physical and mental health (Qu et al., 2023), people still have insufficient awareness of its specific influence, particularly concerning academic performance. In the limited existing literature in this field, researchers have primarily explored the influence of short video addiction on factors such as learning motivation (Ye et al., 2022), achievement motivation (Nong et al., 2023), and academic procrastination (Xie et al., 2023). However, variables such as academic anxiety and academic engagement, both of which are regarded as important antecedents of academic performance, have received insufficient research attention (e.g., Chambel and Curral, 2005; Hossain et al., 2021). Furthermore, mindfulness has garnered attention in recent years (Shapiro et al., 2006), with some researchers noting its significant role in the academic domain. Thus, this study explored the influence of short-form video app addiction on academic anxiety and engagement as well as the mediating role of mindfulness in these relationships.

## 2 Literature review and research hypotheses

### 2.1 Short-form video app addiction and academic anxiety

The term “academic anxiety” is used to describe feelings of nervousness and worry in an academic context (Martin, 2007). Pekrun (2006) proposed that academic anxiety includes negative emotional experiences such as restlessness, neuro-emotions, worry, avoidance motivation, and anxious facial expressions. Previous studies have found that academic anxiety has predictive effects on many academic outcomes, such as learning strategies (i.e., memorization, elaboration, and personal best goals; Collie et al., 2017) and self-efficacy (Muris, 2002). Research has further shown that several other factors also influence academic anxiety, such as a personal belief in a just world, task importance, and academic self-efficacy (Na and Tian, 2023; Nie et al., 2011).

Regarding the association between short-form video app addiction and academic anxiety, Enez Darcin et al. (2016) found that smartphone addiction and academic anxiety are significantly positively correlated. Specifically, people frequently encounter sensations of loss, irritability, restlessness, and anxiety when they choose not to use smartphones or have external limitations placed on their smartphone use. Zhang and Zeng (2024) also indicated that smartphone addiction has a significantly positive influence on academic anxiety among college students. In terms of specific disciplines, Zhou et al. (2022) found that the degree of smartphone addiction could influence mathematics anxiety. Thus, based on previous studies, we propose the following hypothesis:

**Hypothesis 1**: Short-form video app addiction has a significantly positive effect on academic anxiety.

### 2.2 Short-form video app addiction and academic engagement

Academic engagement is a state of engagement in learning, while engagement refers to a positive and fulfilling, work-related mental state characterized by vigor, dedication, and absorption (Schaufeli et al., 2002) that encompasses every facet of a student’s educational experience, such as class attendance, assignment completion, interactions with classmates, and outside influences (Schoffstall et al., 2013). Students can face difficulties with academic engagement regardless of their academic abilities or the educational institution they attend (Schoffstall et al., 2013). Previous research has identified several factors that influence academic engagement, such as academic stereotype threat, academic self-efficacy, academic hope, and academic buoyancy (Azadianbojnordi et al., 2020; Bao et al., 2023).

Regarding the correlation between short-form video app addiction and academic engagement, Zhang and Zeng (2024) found that smartphone addiction can significantly influence future academic engagement. Schoffstall et al. (2013) proposed that one potential measure for identifying academic engagement could be the amount of time students dedicate to completing assignments and other coursework outside class. This further implies that, as individuals spend excessive amounts of time on short-form video apps, the time they spend on academic pursuits will decline. Thus, based on the previous research above, we propose the following hypothesis:

**Hypothesis 2**: Short-form video app addiction significantly negatively affects academic engagement.

### 2.3 Mindfulness as a mediator between short-form video app addiction and academic anxiety/engagement

Mindfulness is defined as the awareness that emerges when providing purposeful, nonjudgmental attention to the unfolding of experience in the present moment (Kabat-Zinn, 2003). This psychological construct has received much research attention in recent years (Shapiro et al., 2006), and is regarded as an extremely important factor that affects individual physical and mental health (Xiang et al., 2023).

Previous studies have found that mobile phone addiction has a significant negative predictive effect on mindfulness (Xiang et al., 2023), and several scholars using various research methods have obtained evidence of this relationship. For example, based on the results of a questionnaire, Zhang et al. (2021) posited that mobile phone addiction influences mindfulness. Similarly, Xiang et al. (2023) used the diary method to find results suggesting that mobile phone addiction impacts mindfulness.

Previous studies have also found that mindfulness can negatively predict academic anxiety. For example, Priebe and Kurtz-Costes (2022) found that increased mindfulness is associated with decreased test anxiety. Abedi et al. (2023) reported that mindfulness training has a significant effect on reducing students’ academic anxiety. As a form of awareness characterized by “non-judgment,” mindfulness allows individuals to confront their academic situations with equanimity. This includes the ability to acknowledge one’s own state and understand its true nature as well as accept it, even if it is less than ideal. From this perspective, we believe that the acceptance and affirmation of one’s current state inherent in mindfulness can help reduce academic anxiety. Furthermore, as mindfulness involves wholehearted attention to the present moment, it undoubtedly contributes to individuals dedicating more time and energy to learning, thereby exhibiting increased academic engagement. Several studies (e.g., Liu et al., 2022; Miralles-Armenteros et al., 2019; Mostafavi et al., 2023) have also revealed with fairly high consistency that mindfulness is a reliable positive predictor of engagement.

Thus, we proposed the following hypotheses based on the abovementioned research:

**Hypothesis 3**: Mindfulness mediates the relationship between short-form video app addiction and academic anxiety.

**Hypothesis 4**: Mindfulness mediates the relationship between short-form video app addiction and academic engagement.

### 2.4 The present study

**Figure 1:**
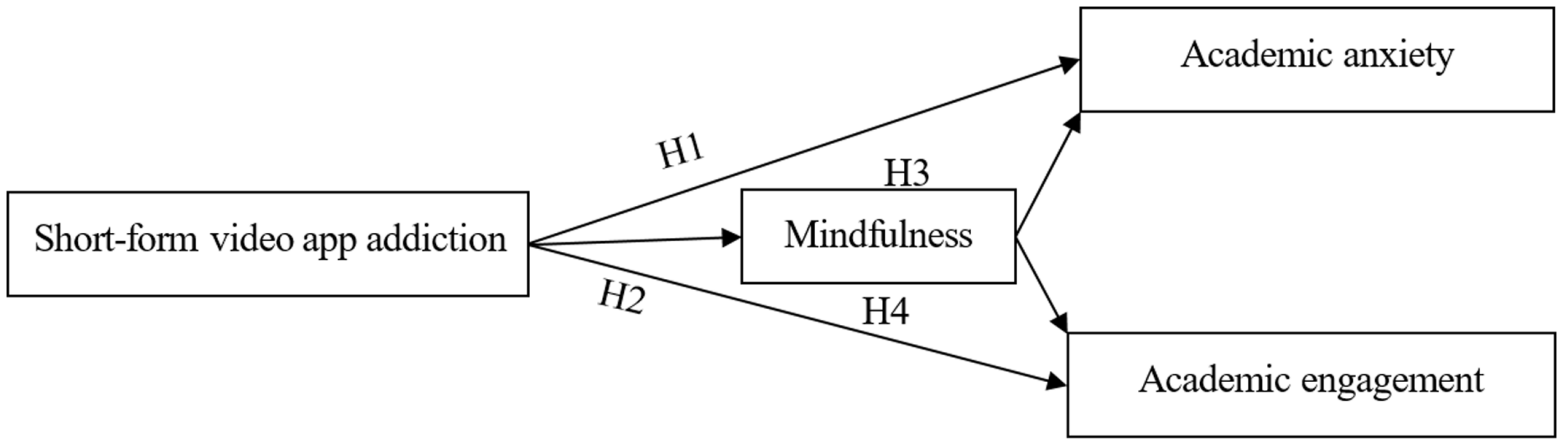
Theoretical model proposed.

This study investigated the effects of short-form video app addiction on academic anxiety and engagement as well as the mediating role of mindfulness in these relationships. Additionally, to explore this theme more effectively, this study specifically concentrated on college students as the participants. This is mainly because, compared with elementary and secondary school students who are more closely supervised, college students generally own smartphones and have more freedom to manage their time. Previous studies have also indicated that young adults are the primary users of short video apps (Qu et al., 2023) and are thus more likely to become addicted to them, making them reasonable research participants (Figure 1).

## 3 Method

### 3.1 Participants

After excluding 463 participants for poor response quality, 1,879 valid questionnaires were collected, with an effective response rate of 80.23%. Detailed demographic information of the participants is shown in Table 1.

**Table 1:** Demographic information of participants.

		Number	Percentage	Demographic variable		Number	Percentage
Gender	males	689	36.70%	Monthly household incomes (RMB)	< 2,000	208	11.10%
	females	1,190	63.30%		2,001–3,000	220	11.70%
Age	≤18	341	18.10%		3,001–4,000	247	13.10%
	19	747	39.80%		4,001–5,000	224	11.90%
	20	500	26.60%		5,001–6,000	226	12.00%
	21	186	9.90%		6,001–7,000	174	9.30%
	22	76	4.00%		7,001–8,000	122	6.50%
	≥23	29	1.50%		8,001–9,000	99	5.30%
					9,001–10,000	100	5.30%
					> 10,000	259	13.80%

RMB is the official currency of China.

### 3.2 Data collection tools

This study collected participants’ personal information and assessed their short video addiction status, mindfulness levels, academic anxiety, and academic engagement levels. The tools and methods employed to collect these data are described below.

#### 3.2.1 Personal information

Participants’ demographic characteristics were collected using a personal information form developed by the researcher, which included gender, age, and monthly household income. The section for collecting personal information was placed at the beginning of the questionnaire, and participants were required to complete this section before proceeding to the formal questionnaire content.

#### 3.2.2 Short-form video app addiction

The short-form video app addiction scale (Choi and Lim, 2016; Zhang et al., 2019) was used to measure participants’ short-form video app addiction levels. The six items were rated on a 7-point Likert scale ranging from 1 (almost never) to 7 (almost always). A sample item is as follows: “My family or friends think that I spend too much time on short-form video apps.” Higher scores indicate higher levels of short-form video app addiction. In this study, Cronbach’s alpha coefficient was 0.89 for this scale.

#### 3.2.3 Mindfulness

The Chinese version of the Mindful Attention Awareness Scale (MAAS; Deng et al., 2012) was used to measure mindfulness. Five items were rated on a 6-point Likert scale ranging from 1 (almost always) to 6 (almost never). A sample item is as follows: “I forget a person’s name almost as soon as I’ve been told it for the first time.” The higher the score, the higher the mindfulness level. In this study, Cronbach’s alpha coefficient was 0.84 for this scale.

#### 3.2.4 Academic anxiety

The Academic Anxiety Sub-questionnaire of the Academic Emotions Questionnaire was used to measure academic anxiety (Dong and Yu, 2007). The seven items were rated on a 5-point Likert scale ranging from 1 (strongly disagree) to 5 (strongly agree). A sample item is as follows: “I’m very worried that my grades are worse than others.” The higher the score, the higher the level of academic anxiety. In this study, Cronbach’s alpha coefficient for the questionnaire was 0.89.

#### 3.2.5 Academic engagement

Engagement scales were used to measure academic engagement (Fang et al., 2008; Schaufeli et al., 2002). This scale had 17 items rated on a 7-point Likert scale ranging from 1 (extremely disagree) to 7 (extremely agree). The scale included three dimensions: vigor (e.g., “When I get up in the morning, I feel like going to class”), dedication (e.g., “My study inspires me”), and absorption (e.g., “Time flies when I am studying”). Higher scores indicated higher levels of academic engagement. In this study, Cronbach’s alpha coefficient was 0.94 for this scale.

### 3.3 Procedure and data analysis

Data were collected primarily using Wenjuanxing, a popular online survey platform in China, in April 2024. Convenience sampling was used, which mainly included college students from Hunan, China. The specific collection process involved sending the questionnaire as a link to the participant groups, and the participants autonomously decided whether to complete the questionnaire. Data from completed questionnaires were automatically saved and served as the foundation for the data analysis process.

After receiving the questionnaire data, the researchers first conducted quality checks and removed questionnaires that did not meet quality standards. Then, SPSS 20.0 was used for descriptive analysis, reliability analysis, validity analysis, common method variance, and correlation analysis of short-form video app addiction, mindfulness, academic anxiety, and academic engagement. Finally, structural equation modeling (SEM) was conducted with AMOS 24.0, to determine the effect of short-form video app addiction on academic anxiety and engagement and the mediating role of mindfulness in these relationships. Considering the significant differences in gender, age, and family monthly income dimensions for the two dependent variables, (i.e., academic anxiety and engagement), they were treated as control variables in the data analysis.

## 4 Results

### 4.1 Preliminary analyses

Some important preliminary analyses were conducted. First, skewness and kurtosis were calculated for all continuous variables. Kurtosis and skewness were between −0.92 and 1.39 and between −0.65 and 0.26, respectively, indicating the shape of the data distribution in the study may be normal based on the standard of kurtosis ≤10.0 and skewness ≤3.0 (Kline, 2015). Second, the CR of short-form video app addiction, mindfulness, academic anxiety, and academic engagement (0.88, 0.85, 0.89, and 0.96, respectively) were greater than 0.70, and their average variance extracted (AVE) values (0.55, 0.53, 0.53, and 0.89, respectively) were greater than 0.50. The square root of the AVE on the diagonal of the scale was mostly higher than the Pearson correlation among the scales, indicating that these variables were reliable and valid based on standard values (Fornell and Larcker, 1981; Hair et al., 1998). Third, common method variance (CMV) was measured using two methods. The results of Harman’s single-factor test (Podsakoff et al., 2003) showed that the first factor extracted only 30.03% of the variance, indicating that none of the other factors accounted for the majority of the variance. The result of single-factor confirmatory analysis with all measurement items loading on a common latent factor showed that chi-square normalized by degrees of freedom (*χ*^2^/df) = 32.18, goodness-of-fit index (GFI) = 0.51, comparative fit index (CFI) = 0.53, normed fit index (NFI) = 0.49, incremental fit index (IFI) = 0.53, and root mean square error of approximation (RMESA) = 0.13, which indicated a poor model fit based on relevant standard. Both approaches demonstrate that CMV is not a major concern.

The means, standard deviations, and correlations among the four variables were measured. As Table 2 shows, short-form video app addiction was significantly negatively correlated with mindfulness and academic engagement, which was significantly positively correlated with academic anxiety. Mindfulness was significantly and negatively correlated with academic anxiety and significantly positively correlated with academic engagement. Academic anxiety was significantly negatively correlated with academic engagement.

**Table 2:** Descriptive statistics and correlations.

	M	SD	SVA	Mindfulness	AA	AE
SVA	3.31	1.08	0.74			
Mindfulness	3.73	0.97	−0.33^***^	0.73		
AA	3.35	0.86	0.27^***^	−0.22^***^	0.73	
AE	3.96	0.89	−0.29^***^	0.37^***^	−0.05^*^	0.94

SVA, Short-form video app addiction; AA, Academic anxiety; AE, Academic engagement. ^*^p < 0.05, ^**^p < 0.01, ^***^p < 0.001. The data on the diagonal of the different questionnaires represent the square root of the AVE. The data below the diagonal represent the correlation coefficients between the different questionnaires.

### 4.2 Measurement model

A measurement model was constructed and tested for the interrelationships among short-form video app addiction, mindfulness, academic anxiety, and academic engagement. A CFA of the measurement model was conducted to examine whether it provided an adequate fit to the data. The result showed that *χ*^2^/df = 4.98, GFI = 0.91, CFI = 0.93, TLI = 0.93, NFI = 0.92, RMSEA = 0.05, and SRMR = 0.06, denoting the measurement model showed a good fit to the data.

### 4.3 Structural model

The structural model was then tested, *χ*^2^/df = 4.95, GFI = 0.91, CFI = 0.93, TLI = 0.93, NFI = 0.92, RMSEA = 0.05, and SRMR = 0.06, and showed good fit to the data.

Short-form video app addiction had significant total and direct effects on academic anxiety (β = 0.26, SE = 0.03, *p* < 0.001, 95% CI [0.20, 0.31]; β = 0.21, SE = 0.03, *p* < 0.001, 95% CI [0.14, 0.27], respectively); thus, Hypothesis 1 was supported. Short-form video app addiction had significant direct effects on academic engagement (β = −0.27, SE = 0.03, *p* < 0.001, 95% CI [−0.34, −0.21]; β = −0.13, SE = 0.04, *p* < 0.001, 95% CI [−0.20, −0.06], respectively); thus, Hypothesis 2 was supported.

Short-form video app addiction significantly and negatively predicted mindfulness (β = −0.37, SE = 0.03, *p* < 0.001, 95% CI [−0.43, −0.31]), and mindfulness significantly and negatively predicted academic anxiety (β = −0.13, SE = 0.03, *p* < 0.001, 95% CI [−0.20, −0.07]); thus, Hypothesis 3 was supported. Furthermore, the 95% CIs for the indirect effect of short-form video app addiction on academic anxiety via mindfulness (0.03, 0.08) did not include zero, indicating a significant mediating relationship. The mediation path model explained 19.23% of the variance in academic anxiety.

**Figure 2:**
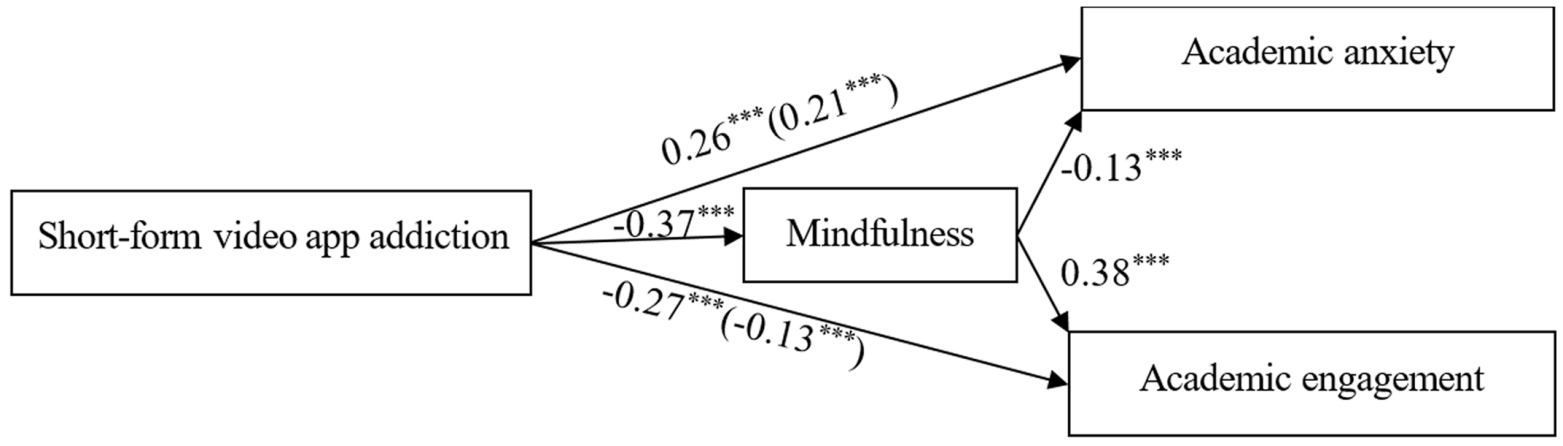
Structural model. *p < 0.05, **p < 0.01. The numbers in parentheses is the value of direct effect.

Mindfulness significantly and positively predicted academic engagement (β = 0.38, SE = 0.03, *p* < 0.001, 95% CI [0.32, 0.45]). After combining the relationship between short-form video app addiction and mindfulness, Hypothesis 4 was supported. Furthermore, the 95% CIs for the indirect effect of short-form video app addiction on academic engagement via mindfulness (−0.18, −0.11) did not include zero, further indicating the presence of a significant mediating relationship. The mediation path model explained 51.85% of the variance in academic engagement. All indicators are listed in Figure 2 and Table 3.

**Table 3:** Differential effects from the structural model.

Path	Types	Estimate	SE	Lower	Upper	*p*
SVA → AA	Direct	0.21	0.03	0.14	0.27	<0.001
	Indirect	0.05	0.01	0.03	0.08	<0.001
	Total	0.26	0.03	0.20	0.31	<0.001
SVA → AE	Direct	−0.13	0.04	−0.20	−0.06	<0.001
	Indirect	−0.14	0.02	−0.18	−0.11	<0.001
	Total	−0.27	0.03	−0.34	−0.21	<0.001

## 5 Discussion

This study examined the effect of short-form video app addiction on academic anxiety and engagement, as well as the mediating role of mindfulness, in a population of college students. The research findings indicate that short-form video app addiction significantly positively predicts academic anxiety and significantly negatively predicts academic engagement. Furthermore, mindfulness has a significant mediating role in the relationships between short-form video app addiction and both academic anxiety and academic engagement.

Our results support the basic assumption that short-form video app addiction significantly and positively predicts academic anxiety, showing that participants who experienced more short-form video app addiction were more likely to report high levels of academic anxiety, which is in line with previous studies (e.g., Enez Darcin et al., 2016; Zhang and Zeng, 2024; Zhou et al., 2022). A possible explanation for this finding is that, in the excessive use of short video apps, students waste a considerable amount of time to the extent that they are unable to dedicate sufficient time to their academic pursuits. This results in their potential inability to pass various assignments, exams, and so on, which directly affects their eligibility to obtain a college diploma. Consequently, they experience heightened academic anxiety. Academic failure can damage an individual’s self-esteem, especially in China, where academic achievement is emphasized. This also implies that when students face difficulty passing academic tests because they are engrossed in short-form video apps, their academic anxiety may increase.

Short-form video app addiction significantly negatively predicted academic engagement, indicating that participants who experienced more short-form video app addiction were less likely to report high levels of academic engagement, which is consistent with many previous studies (e.g., Zhang and Zeng, 2024; Zhen et al., 2020). One possible explanation for this outcome is time, which is also the focal point of the definition of short-form video addiction (e.g., Zhang et al., 2019). When individuals become addicted to short-form video apps, they allocate a significant amount of time. However, when an individual’s time is limited, the time they invest in academics decreases significantly; that is, their academic engagement decreases. Another possible explanation for this outcome is attention. Some studies suggest that addicted users may experience more difficulties in maintaining attention (Chen et al., 2023), be more susceptible to distractors, and have more difficulties in inhibiting the influence of distractors and completing tasks (Chen et al., 2023). These attention-related issues clearly reduced individual academic engagement.

In support of our hypotheses, mindfulness may mediate the relationship between short-form video app addiction and academic anxiety. Specifically, students with higher short-form video app addiction showed lower mindfulness, which increased their academic anxiety. This can be attributed to the following factors. First, since the total amount of time is fixed, spending too much time on the app will reduce an individual’s time on mindfulness practice. Furthermore, when individuals are addicted to short-form video apps, they only need to exert minimal cognitive effort to input a large amount of information into their minds, which can easily lead to cognitive inertia (Huang and Leung, 2009; Zhang et al., 2023), thus hindering the improvement of mindfulness. Second, Mindfulness is characterized by an individual’s non-judgmental awareness of the present moment, requiring them to maintain a state of experiential presence. This involves not only experiencing the joy of learning but also realistically perceiving the challenges and emotions associated with academic pursuits, such as anxiety. However, due to the non-judgmental nature of mindfulness, anxiety is not amplified excessively but remains within a manageable range. In the context of addiction to short video applications, mindfulness levels tend to be lower. This suggests a decreased capacity for non-judgmental awareness of the academic process, indicating that there is room for an increase in academic anxiety.

Mindfulness may act as a mediator in the relationship between short-form video app addiction and academic engagement, which validates our hypotheses. Specifically, students with higher levels short-form video app addiction showed lower levels of mindfulness, which would in turn lower their academic engagement. The previous section explained why short-form video app addiction influences mindfulness. Therefore, only the reasons why mindfulness affects academic engagement will be explained.

According to Langer (2016) and Zhang et al. (2017), in a state of mindfulness, individuals direct their attention to the object of present’ observation, and interested in different ways of exploring information and acquiring knowledge, and thus actively engages in the learning process. Whenever their thoughts wander, attention is gently and steadfastly redirected back to the original focal point. Liu et al. (2022) also indicated that this is represented in tasks involving fun learning which possibly allows learners to have a higher sense of comfort and lower fear and pleasure while studying. Students with mindfulness will have greater fun with the process of learning, which makes them engaged and have higher learning functions. Thus, students with lower levels of mindfulness due to addiction to short-form videos may struggle to fully enjoy the learning process and lack the motivation and awareness to acquire information and knowledge, consequently leading to lower academic engagement.

## 6 Implications and limitations

This study identified the relationship between short-form video app addiction and academic anxiety and engagement as well as the mediating role of mindfulness in this relationship, which is of great practical significance. Two measures should be taken to mitigate the negative effects of short-form video app addiction on academic anxiety and engagement. First, as evidenced by this study, short-form video app addiction was the most direct factor influencing academic anxiety and engagement. Therefore, society, families, and schools should seriously consider the issue of short video addiction and recognize its significant harm to students’ academic performance. This can be addressed by enhancing individual awareness among college students, limiting the usage time of short-form video apps, implementing various measures to control this phenomenon, and ensuring that students’ use of short videos remains within reasonable limits. Second, as indicated in this study, mindfulness serves as a mediating variable between short-form video app addiction, academic anxiety, and engagement. Therefore, schools should adopt appropriate measures. Specifically, schools can help students understand the concept and significance of mindfulness through courses and training, enabling them to master its core principles, thereby enhancing their level of mindfulness. Ultimately, this intervention could be a key factor in reducing short video addiction, alleviating academic anxiety, and increasing academic engagement. According to previous studies, suddenly stopping to watch short videos could lead to more negative emotions, such as anxiety and depression, in college students. After an individual stops watching short videos, the fear of missing out may arise (Wang et al., 2023). Therefore, proceeding gradually rather than abruptly discontinuing is important when addressing short video app addiction.

This study has several limitations that should be considered and modified in the future. First, the self-report measures used in this study were subject to response bias (e.g., social desirability effects). For example, the participants may have perceived short-form video app addiction as disgraceful. Therefore, certain methods should be used in combination in future studies. Second, this study adopted a cross-sectional design, which does not allow for inferences regarding causal relationships. Longitudinal studies are required to further examine the relationships between these variables. Third, the four variables (short-form video app addiction, mindfulness, academic anxiety, and academic engagement) involved in this study only focused on individual circumstances, with insufficient attention paid to objective and environmental variables despite their importance to the aforementioned variables. For example, previous research has indicated a relationship between peer communication, parental phubbing, and short-form video app addiction (Wang and Lei, 2022). Future studies should investigate the influence of these factors on the variables examined in this study.

## 7 Conclusion

The negative impact of short-form video app addiction on academic performance has gradually attracted increasing attention. Against this backdrop, this study investigated the influence of short-form video app addiction on academic anxiety and engagement among college students, as well as the mediating role of mindfulness in these relationships. Despite some limitations, this study’s overall findings provide helpful insights for schools, families, and society overall for understanding the detrimental effects of short-form video app addiction and taking measures to alleviate it, enhance mindfulness, and promote academic performance.

## Data Availability

The raw data supporting the conclusions of this article will be made available by the authors, without undue reservation.

## Footnotes

^1^Data Source: TikTok Mobile Application Release.
